# Increased Serum Levels of Tumor Necrosis Factor-Alpha, Resistin, and Visfatin in the Children with Autism Spectrum Disorders: A Case-Control Study

**DOI:** 10.1155/2016/9060751

**Published:** 2016-11-28

**Authors:** Mohammad Ali Ghaffari, Elham Mousavinejad, Forough Riahi, Masoumeh Mousavinejad, Mohammad Reza Afsharmanesh

**Affiliations:** ^1^Biochemistry Department, Medical School, Ahvaz Jundishapur University of Medical Sciences, Ahvaz, Iran; ^2^Cellular and Molecular Research Center, Ahvaz Jundishapur University of Medical Sciences, Ahvaz, Iran; ^3^Department of Psychiatry, Medical School, Ahvaz Jundishapur University of Medical Sciences, Ahvaz, Iran; ^4^Centre for Stem Cell Biology (CSCB), Department of Biomedical Science, The University of Sheffield, Sheffield, UK; ^5^Hyperlipidemia Research Center, Ahvaz Jundishapur University of Medical Sciences, Ahvaz, Iran

## Abstract

*Background*. Autism spectrum disorders (ASDs) are complex disorders where the pathogenesis is not fully understood. Several proinflammatory and immunoinflammatory disturbances have been observed in the etiology of ASD. There is, however, limited knowledge on variations of adipokines in ASD. The present study aimed to analyze the serum levels of resistin, visfatin, and tumor necrosis factor-alpha (TNF-*α*) in children with ASD in relation to body weight, gender, and ASD severity level.* Method*. In total, 30 children with ASD (mean age: 7.72 ± 2.65 y; range; 4–12 y) and 30 healthy children (mean age: 8.4 ± 2.66 y; range: 4–12 y), including males and females, were matched for age, gender, and body mass index (BMI). Serum samples were collected, and visfatin, resistin, and TNF-*α* serum levels were measured using an enzyme-linked immunosorbent assay (ELISA) kit.* Result*. Serum visfatin, resistin, and TNF-*α* levels in children with ASD were significantly higher than that in the healthy patients (*p* < 0.05). Two significant correlations were found: a correlation between resistin and visfatin with TNF-*α* in children with ASD (*R* = 0.8 and* R* = 0.62, resp.) and a correlation between resistin and visfatin in children with ASD (*R* = 0.66).* Conclusion*. Higher TNF-*α*, resistin, and visfatin levels were found in children with ASD in comparison with controls, suggesting that elevated levels of serum proinflammatory agents may be implicated in the pathophysiology of ASD.

## 1. Introduction 

Autism spectrum disorders (ASDs) are pervasive neurodevelopmental disorders characterized by clinical impairments that result in deficits in interacting with the environment and social, individual, and behavioral skills [1]. In psychiatry, ASDs are characterized by using the Autism Diagnostic Interview, Revised (ADI-R), which lists impairments in social communications and interactions, repetitive behaviors, and restricted interests as characteristics of the disorder. ASDs occur in children under 3 years of age, and its prevalence is four to five times greater in males [2, 3]. The prevalence of ASD in the United States was 4-5 in 1,000 in 1990, 1 in 150 in 2007, 1 in 91 in 2009, and 1 in 50 in 2013 [4–6]. Therefore, the mechanisms underlying this disorder need to be elucidated in order to create better treatment options for people with ASD.

ASDs have a complex neurobiological basis that is not fully clarified [1]. The cause of ASD is unknown, and its etiology is poorly understood. Moreover, no biomarkers have been identified in ASD, nor has any treatment approach [7, 8]. Several disturbances in proinflammatory and immunoinflammatory factors have been observed in ASDs. Although the precise mechanism underlying the pathophysiology of ASD is yet to be identified, accumulating evidences suggest that the abnormality of inflammatory factors may be implicated [9]. Recent studies report alterations in the immunoinflammatory system of individuals with ASDs [10]. The immune system produces and releases a variety of immunoinflammatory and proinflammatory factors including the adipokines such as visfatin, resistin, leptin, and adiponectin, cytokines, and chemokines such as tumor necrosis factor-alpha (TNF-*α*) and interleukin-6 (IL-6) [11]. Adipokines act as intermediates of metabolic activity and also function as immunomodulators of distinctive and adaptive immune cells such as monocytes and macrophages [12]. Adipokines contribute to adverse metabolic and immune responses by stimulating lipid gathering and proinflammatory cytokine production in cells [13].

Studies of brain tissue and cerebrospinal fluid from postmortem individuals with ASD revealed high levels of TNF-*α*, IL-6, and interleukin-1-beta (IL-1-*β*) [14]. TNF-*α*, IL-6, IL-1, and interleukin-12 (IL-12) are significant inflammatory factors acting through nuclear factor kappa-light-chain-enhancer of activated B cells (NF-*κβ*) to increase the expression of resistin [15]. NF-*κβ* is a complex protein which regulates DNA replication in response to immunoinflammatory activities [16]. Resistin, a proinflammatory cytokine, has been implicated in the pathogenesis of several inflammatory central nervous system disorders [17]. Chumakov et al. reported the contribution of resistin to chemotaxis of leukocytes, via upregulating the gene expression of intracellular adhesion molecule-1 and vascular cell-adhesion molecule-1 [18]. Resistin and visfatin, which are secreted from adipose tissues, macrophages, and monocytes, are considerably important owing to their roles in metabolic functions and immunoinflammatory system [19]. Visfatin with the molecular weight of 52 kDa is an adipokine, which is secreted from visceral fat tissue and monocytes in response to TNF-*α*, IL-6, and IL-1-*β* and has regulatory activities in the immunoinflammatory system [20]. Visfatin has an insulin-like role and its synthesis is regulated by several factors including glucocorticoids, TNF-*α*, and IL-6 [21]. The roles of resistin and visfatin in the pathophysiology of many diseases such as diabetes [22], obesity [23], immunoinflammatory system impairments [24], rheumatoid arthritis [25], and cardiovascular diseases [26] have been demonstrated. For ASD, however, there is limited data available related to variation and significance of adipokines in ASDs. In addition, resistin and visfatin may also be involved in ASDs pathogenesis. To the best of our knowledge, there are a few studies that have shown alterations in the adipokines in ASDs. Considering the immunoinflammatory function of resistin and visfatin, we hypothesized that peripheral resistin and visfatin levels may be increased in individuals with ASD. In the present study, we examined serum levels of resistin, visfatin, and TNF-*α* in children with ASD in comparison with body mass index- (BMI), sex-, and age-matched control subjects. Furthermore, the relationship between serum levels of adipokines and the severity of ASD was investigated.

## 2. Methods

### 2.1. Participants

This study was performed as a case-controlled report between June 2013 and March 2014. In total, 30 children diagnosed with mild to severe ASD (*N* = 30; mean age, 7.72 ± 2.65 y; range, 4–12 y) were enrolled into the study. Healthy children (*N* = 30; mean age, 8.4 ± 2.66 y; range, 4–12 y) were also selected and diagnosed by a pediatric endocrinologist. All of the subjects (both ASD and control groups) had normal karyotype without comorbidities, such as diabetes and cardiovascular diseases. They were screened for comorbid psychiatric illnesses (schizophrenia, affective disorders, mental retardation, and personality or behavioral disorders) using the structured clinical interview for DSM-IV (SCID) [27] and for other diseases, such as common cold and asthma. Children in the ASD group were drug naive and had supplementation or been free of psychoactive medications for at least 3 months. All children were Iranian, born and living in the state of Khuzestan. The study was approved by the ethics committee of the Medical University of Medical Sciences, Ahvaz, Iran. Written consent was also obtained from the parents of the children.

### 2.2. Structured Clinical Interview

ASD was diagnosed according to the Diagnostic and Statistical Manual of Mental Disorders, Fourth Edition, Text Revision (DSM-IV-TR), American Psychiatric Association [28], Autism Diagnostic Observation Schedule (ADOS), and Childhood Autism Rating Scale (CARS) [29]. According to the scores [30], 18 children were classified as having mild autism (Asperger syndrome) and the remaining 12 as having severe autism (autistic disorder). The ASD and control groups were followed up in the Departments of Child Psychiatry and Department of Pediatrics of the University Hospital of Golestan, Ahvaz, Iran, respectively.

### 2.3. Specific Measurements

#### 2.3.1. Anthropometric Measurements

All of the participants were measured for weight, height, and waist circumference as well as their BMI, which was calculated as the ratio of body weight (kg) to height squared (m^2^). A standard deviation (SD) score for BMI (BMI-SDS) was also calculated according to the current Iranian population normal range [31].

### 2.4. TNF-*α*, Visfatin, and Resistin Assays

Fasting blood samples were collected in the morning (between 8.00 AM and 10.00 AM). The serum was separated from blood by centrifugation at 1300 xg for 10 minutes. Serum samples were stored at −20°C. Concentrations of TNF-*α*, resistin, and visfatin were measured using commercial ELISA kits (Bioassay Technology Laboratory) according to the corresponding manufacturer's protocol and by ELIZA reader (Bio-Tek model, USA). Assay data were analyzed using GEN-5 Software (v.2.01.14, Bio-Tek Instruments, Winooski, VT, USA). The sensitivity values for visfatin, resistin, and TNF-*α* were 0.25 ng/mL, 10.2 ng/mL, and 1.52 ng/mL, respectively. In addition, the intra- and interassay errors for these assays were less than 10% and 12%, respectively.

### 2.5. Statistical Analysis

Data were reported as mean ± SD. All statistical analyses were performed using SPSS software (version 16) with significance level set at *p* < 0.05 and group differences analyzed using Student's* t*-test. Evaluation of the relationships between serum levels of TNF-*α*, visfatin, and resistin and clinical variables among the subjects (ASD and control groups) was performed with Pearson's correlation coefficient. Correlations were calculated using the Spearman rank test. Accuracy of the diagnostic resistin, visfatin, and TNF-*α* measurement tests was assessed via curve analysis of the receiver operating characteristic (ROC).

## 3. Results

Weight, height, and BMI were calculated for all participants. The mean ± SD of these variables, as well as age, for ASD and control groups is presented in Table 1. There were no significant differences in the mean and distribution of age, weight, height, and BMI between the two groups.

### 3.1. The Comparison of TNF-*α*, Visfatin, and Resistin Serum Levels between ASD and Control Groups

Mean serum levels of TNF-*α*, visfatin, and resistin for all participants are shown in Table 2. Statistical analyses revealed significant differences between the two groups in all measured factors. TNF-*α* was higher in the ASD group in comparison with controls; this was also true for resistin (Table 2).

### 3.2. The Comparison of Ratios of TNF-*α*, Visfatin, and Resistin to BMI between ASD and Control Groups

As serum levels of resistin and visfatin depend on the amount of adipose tissue, adipokine levels were adjusted by dividing the measured concentration by BMI. Mean ratios of adipokine serum levels to BMI are shown in Table 3. The ratios of resistin, visfatin, and TNF-*α* levels to BMI were significantly higher in the ASD group than in the control group.

### 3.3. Correlations between Adipokines and Clinical or Anthropometric Parameters

Correlations between serum levels of visfatin, resistin, and TNF-*α* are presented in Table 4. TNF-*α* serum levels positively correlated with both resistin and visfatin. In addition, we observed a correlation between proinflammatory factors and the amount of resistin and visfatin. The relationship between age and serum levels of adipokines in the ASD group was examined using Pearson's correlation coefficient test. The relationship between adipokine serum levels and ASD severity (autistic disorder or Asperger syndrome) was also examined using Pearson's correlation coefficient where no significant correlation was observed.

**Figure 1:**
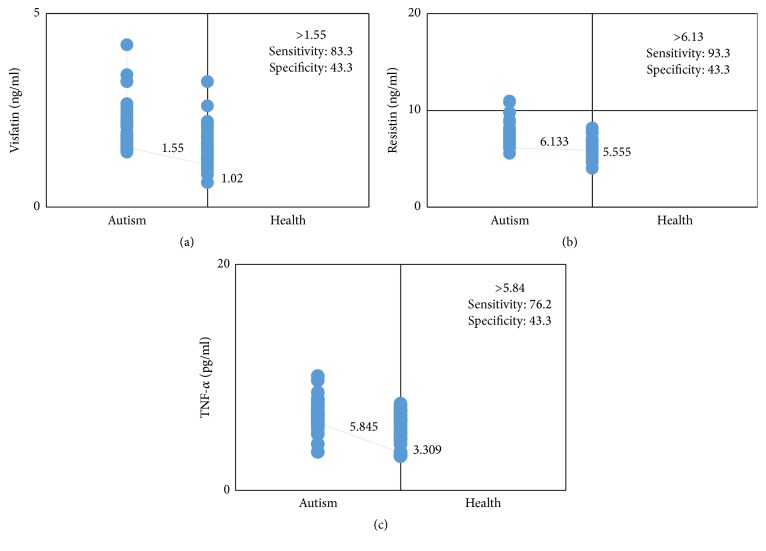
Interactive dot diagram comparing levels of visfatin (a), resistin (b), and TNF-*α* (c) in autistic and healthy children.

**Figure 2:**
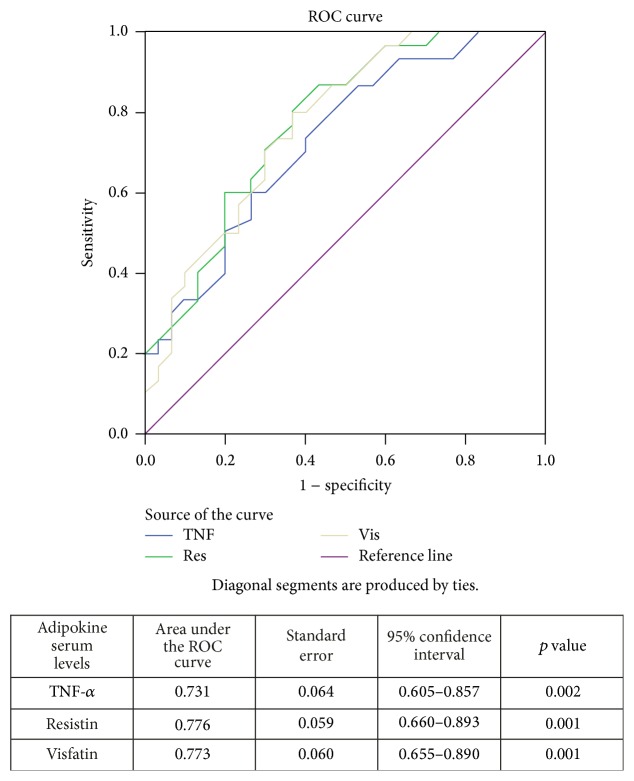
Comparison of receiver operating characteristic (ROC) curves for TNF-*α*, resistin, and visfatin in autistic and healthy children.

In order to assess the diagnostic value of adipokine serum levels for children with ASDs from all examined cases, a ROC analysis was performed. Based on ROC analysis, the cutoff value, sensitivity, and specificity of visfatin in the ASD group were 1.55 ng/mL, 83.3%, and 43.3%, respectively (Figure 1(a)). For resistin, the cutoff value was 6.13 ng/mL, sensitivity was 93.3%, and specificity was 43.3% in the ASD group (Figure 1(b)). The values for TNF-*α* in the ASD group were 5.84 ng/mL, 76.2%, and 43.3%, respectively (Figure 1(c)). The area under the ROC curve for visfatin was 0.773, for resistin was 0.776, and for TNF-*α* was 0.731, indicating that serum levels of these three adipokines are biomarkers differentiating subjects with ASD from healthy children (Figure 2). There was no relationship between adipokine serum levels and ASD severity; thus, this figure indicates that serum levels of TNF-*α*, resistin, and visfatin can be used as differentiating biomarkers in children with ASD.

## 4. Discussion

This study was designed to determine whether serum levels of TNF-*α*, resistin, and visfatin were altered in children with ASD without additional comorbid diseases. To eradicate the possible effect of obesity on the adipokine levels, serum concentrations of adipokines were corrected with the BMI. We demonstrated that children with ASD had elevated adipokine levels and adipokine to BMI ratios (Tables 2 and 3). Resistin and visfatin, which are secreted from fat tissues, macrophages, and monocytes, are known to play significant roles in metabolic and immunoinflammatory functions [32]. Moreover, in spite of having similar fat tissue, there is an alteration in levels of resistin and visfatin between children with ASD and healthy children. The results suggest that these adipokines may be implicated in the immunoinflammatory of ASDs, irrespective of their confirmed role in body weight.

There is limited information about the role of adipokines in ASD. Two studies indicate that elevated adipokine levels might be implicated in the pathophysiology of autism and Rett syndrome [33, 34]. Rodrigues et al. reported low levels of resistin, high levels of leptin, and unaltered levels of adiponectin in the serum of individuals with ASD. Visfatin and resistin can upregulate the production of the proinflammatory cytokines such as IL-1 *β*, TNF-*α*, and IL-6 [35]. Previous studies have reported a correlation between increases in the expression of TNF-*α*, resistin, and visfatin in a variety of inflammatory diseases including rheumatoid arthritis, inflammatory bowel disease [8], and psoriasis [9]. Peltola et al. observed inflammatory changes in the brain tissue of patients with autism using scans [36]. Another study by Jyonouchi et al. showed high levels of TNF-*α*, IL-6, and IL-1-*β* in children with autism [37]. Furthermore, Nehus et al. found a positive correlation between increasing resistin levels and inflammatory cytokines in the serum [38]. Our results are in agreement with this literature; we demonstrated increasing levels of TNF-*α* in the serum of children with ASD and also observed a significant positive correlation between TNF-*α* and resistin and visfatin.

Several studies revealed increases in TNF-*α*, resistin, and visfatin levels in various disorders such as cholelithiasis [39], diabetes, and atherosclerosis [40]. However, no literature about increase of TNF-*α*, resistin, and visfatin in ASDs has been reported. TNF-*α* may act as a key player in the upregulation of resistin and visfatin expression in the cells of patients with ASD. Therefore, our results indicate that immune dysfunctions may be associated with ASDs. Further studies need to be conducted to examine the molecular mechanism(s) of altered immune response in patients with ASDs.

ASDs have been associated with malfunctions of multiple biological systems and pathways, especially the cytokines of the immune system. These changes in immune functions may influence crucial neurodevelopment processes [41] and subsequently the severity level of ASDs. However, because our results do not show alteration of the factors measured between the two ASD groups (autistic disorder and Asperger syndrome), we suggest these adipokines may not affect the severity level of ASDs.

The most interesting result of the present study is that TNF-*α*, resistin, and visfatin are good markers of an ASD diagnosis. The study had a few limitations. Firstly, the sample size was small and does not represent all the children with ASDs. The children were selected according to DSM-IV-TR without comorbid disease and no drug use and supplementation for at least 3 months, which limited the available subject pool. Secondly, the cross-sectional analysis in this study prevented any chronological or cause-effect inferences. Thirdly, we were, however, unable to infer from the results whether the altered adipokine levels play a causative role in ASDs or if they alter a secondary inflammation to a chronic one.

In summary, elevated levels of TNF-*α*, resistin, and visfatin may play a role in the pathophysiology of ASD. Prospective studies with greater number of patients and other adipokine assays need to be undertaken to clarify the precise mechanism of this disorder.

## Figures and Tables

**Table 1:** Demographic and clinical characteristics of autistic children and healthy children.

Characteristics	Children with autism (*n* = 30)	Healthy children (*n* = 30)	*t*-value	*p* value
Age, years, mean ± SE	7.72 ± 2.65	8.4 ± 2.66	−0.99	0.32
Sex, *n*	boys: 22;	boys: 22;		
	girls: 8	girls: 8		
Weight, kg	26.52 ± 8.2	27.26 ± 8.85	−0.33	0.73
Height, cm	126.3 ± 15.56	127.7 ± 16.58	−0.34	0.73
BMI, kg/m^2^	16.15 ± 1.38	16.19 ± 1.38	−0.11	0.90
BMI-SDS	0.34 ± 0.17	0.24 ± 0.15		

BMI: body mass index; BMI-SDS: body mass index-standard deviation scores.

All *p* values are gotten from two-tailed Student's *t*-test.

**Table 2:** Mean values of adipokine serum levels in children autism and healthy children.

Adipokine serum levels	Autistic children	Health children	*t*-value	*p* value
	*n* = 30	*n* = 30		
	Boys/girls (22/8)	Boys/girls (22/8)		
TNF-*α* pg/mL				
All subjects	6.7 ± 1.43	5.38 ± 1.45	3.5	0.001
Boys	6.53 ± 1.43	5.4 ± 1.53	3.5	0.002
Girls	7.16 ± 1.44	5.23 ± 1.25	1.77	0.12
Resistin ng/mL				
All subjects	7.66 ± 1.33	6.27 ± 1.16	4.3	0.000
Boys	7.4 ± 1.26	5.63 ± 1.13	3.5	0.002
Girls	8.2 ± 1.4	6.01 ± 1.28	2.8	0.07
Visfatin ng/mL				
All subjects	2.14 ± 0.66	1.58 ± 0.53	3.59	0.001
Boys	2.04 ± 0.55	1.63 ± 0.55	3.4	0.003
Girls	2.42 ± 0.89	1.44 ± 0.48	1.56	0.162

Data are shown as mean ± standard error. *p* values from *t*-test; *p* < 0.05. Autism versus control.

*p* < 0.001. Autism versus control.

**Table 3:** The adipokine serum levels/BMI ratio in autistic children and healthy controls.

Adipokine serum levels/BMI	Autistic children	Healthy children	*t*-value	*p* value
	*N* = 30	*N* = 30		
	Boys/girls 22/8	Boys/girls 22/8		
TNF-*α* pg/mL/ BMI (kg/m^2^)				
All subjects	0.41 ± 0.1	0.33 ± 0.09	3.8	0.00
Boys	0.41 ± 0.1	0.33 ± 0.09	−1.7	0.09
Girls	0.43 ± 0.1	0.33 ± 0.08	0.29	0.77
Visfatin ng/mL/ BMI (kg/m^2^)				
All subjects	0.13 ± 0.04	0.09 ± 0.03	3.4	0.001
Boys	0.13 ± 0.04	0.1 ± 0.03	−0.89	0.38
Girls	0.14 ± 0.06	0.09 ± 0.03	0.72	0.47
Resistin ng/mL/ BMI (kg/m^2^)				
All subjects	0.47 ± 0.1	0.38 ± 0.07	3.8	0.000
Boys	0.47 ± 0.1	0.39 ± 0.06	−2.16	0.05
Girls	0.49 ± 0.1	0.37 ± 0.09	0.75	0.46

Data are shown as mean ± standard error. *p* values from Student's *t*-test. Autism versus control, *p* < 0.05.

**Table 4:** Correlations of TNF-*α*, resistin, and visfatin serum levels in autistic and healthy children.

Examined significant correlation	*R*	*p*
Autistic children		
Visfatin and TNF-*α*	0.62	0.01
Resistin and TNF-*α*	0.80	0.01
Resistin and visfatin	0.66	0.01
TNF-*α* and age	−0.39	0.03
TNF-*α* and weight	−0.40	0.02
TNF-*α* and BMI	−0.24	0.18
Resistin and age	−0.36	0.04
Resistin and weight	−0.34	0.06
Resistin and BMI	−0.22	0.23
Visfatin and age	−0.28	0.1
Visfatin and weight	−0.3	0.1
Visfatin and BMI	−0.2	0.2
Healthy children		
Visfatin and TNF-*α*	0.33	0.06
Resistin and TNF-*α*	0.40	0.02
Resistin and visfatin	0.58	0.01
TNF-*α* and age	−0.32	0.08
TNF-*α* and weight	0.09	0.6
TNF-*α* and BMI	0.13	0.48
Resistin and age	−0.3	0.25
Resistin and weight	0.08	0.64
Resistin and BMI	0.18	0.33
Visfatin and age	0.36	0.05
Visfatin and weight	0.04	0.82
Visfatin and BMI	0.09	0.63

BMI: body mass index; Spearman's rank test, *p* < 0.05.
